# Performance of a Small Language Model Versus a Large Language Model in Answering Glaucoma Frequently Asked Patient Questions: Development and Usability Study

**DOI:** 10.2196/72101

**Published:** 2026-01-06

**Authors:** Adriano Cypriano Faneli, Rafael Scherer, Rohit Muralidhar, Marcus Guerreiro-Filho, Luiz Beniz, Verônica Vilasboas-Campos, Douglas Costa, Alessandro A Jammal, Felipe A Medeiros

**Affiliations:** 1 Bascom Palmer Eye Institute, University of Miami, 900 NW 17th St, Miami, FL, 33136, United States, 1 305-326-6000; 2 Department of Ophthalmology, Federal University of São Paulo, São Paulo, Brazil

**Keywords:** online health information, ChatGPT4.0, glaucoma, large language model, small language model

## Abstract

**Background:**

Large language models (LLMs) have been shown to answer patient questions in ophthalmology similar to human experts. However, concerns remain regarding their use, particularly related to patient privacy and potential inaccuracies that could compromise patient safety.

**Objective:**

This study aimed to compare the performance of an LLM in answering frequently asked patient questions about glaucoma with that of a small language model (SLM) trained locally on ophthalmology-specific literature.

**Methods:**

We compiled 35 frequently asked questions on glaucoma, categorized into 6 domains, including pathogenesis, risk factors, clinical manifestations, diagnosis, treatment and prevention, and prognosis. Each question was posed to both a SLM using a retrieval-augmented generation framework, trained on ophthalmology-specific literature, and to a LLM (ChatGPT 4.0, OpenAI). Three glaucoma specialists from a single institution independently assessed the answers using a 3-tier accuracy rating scale: poor (score=1), borderline (score=2), and good (score=3). Each answer received a quality score ranging from 3 to 9 points based on the sum of ratings from the 3 graders. Readability grade level was assessed using 4 formulas, such as the Flesch-Kincaid Level, the Gunning Fog Index, the Coleman-Liau Index, and the Simple Measure of Gobbledygook Index.

**Results:**

The answers from the SLM demonstrated comparable quality with ChatGPT 4.0, scoring mean 7.9 (SD 1.2) and mean 7.4 (SD 1.5), respectively, out of a total of 9 points (*P*=.13). The accuracy rating was consistent overall and across all 6 glaucoma care domains. Both models provided answers considered unsuitable for health care–related information, as they were difficult for the average layperson to read.

**Conclusions:**

Both models generated accurate content, but the answers were considered challenging for the average layperson to understand, making them unsuitable for health care–related information. Given the specialized SLM’s comparable performance to the LLM, its high customization potential, lower cost, and ability to operate locally, it presents a viable option for deploying natural language processing in real-world ophthalmology clinical settings.

## Introduction

Recent progress in natural language processing (NLP) has been observed in health care, showcasing innovative approaches to preventive measures, diagnostics, and patient assistance. Specifically, large language models (LLMs) such as ChatGPT (OpenAI) have emerged as prominent tools in the field of ophthalmology and other medical specialties since their introduction in November 2022 [1-3]. The conversational interface of ChatGPT and its unsupervised learning approach, particularly notable in its fourth generation, ChatGPT 4.0, has offered a novel and appealing way for patients to access medical information [4,5]. This trend is underscored by the growing reliance on the internet for health-related information, a phenomenon that has become increasingly common among patients. A survey in the United States revealed that two-thirds of adults turn to the internet for health information, with one-third using it for self-diagnosis [6]. However, despite these advancements and the increasing usage of digital resources for health information, the inability of ChatGPT to provide source citations remains a significant drawback, compromising its reliability and limiting its utility in clinical settings [5,7].

Recent literature has explored the role of LLMs in different ophthalmological scenarios. For example, Cai et al [8] demonstrated strong performance of ChatGPT models in ophthalmology board-style certification questions, underscoring their educational potential in training ophthalmologists. Huang et al [9] showed that ChatGPT’s diagnostic capabilities in glaucoma could sometimes surpass those of ophthalmology residents, emphasizing their clinical utility in differential diagnosis and management. Additionally, Raghu et al [10] identified the potential use of LLMs for diabetic retinopathy risk assessment, although they noted several limitations that restrict clinical deployment.

The substantial number of tasks that LLMs can perform highlights their potential for innovative research; however, the substantial computational demands for customizing these models, which may include over 100 billion parameters, present a significant challenge, making the technology largely unattainable due to computational resource limitations [11]. In this context, small language models (SLMs) have emerged as a practical alternative [12]. These scaled-down models offer advantages in terms of computational efficiency, ease of access, and customizability because they require fewer resources and facilitate deployment in more specific contexts [12]. Their adaptability to specific needs and functions allows for the development of precise and accessible NLP tools by leveraging targeted, high-quality references, demonstrating a promising path for specialized applications [12]. SLM can also be used in a closed local network without an internet connection, which diminishes the concerns about patient privacy and leakage of personal health information.

More recently, the use of retrieval-augmented generation (RAG) frameworks in natural language models has enabled precise query processing and the generation of highly accurate and relevant responses. By encoding and vectorizing documents, RAG allows language models to access external information, extending their knowledge beyond what was available in the training data. Furthermore, by integrating external data, RAG enables natural language models to effectively provide source citations, thereby bolstering the credibility of the generated content [13,14].

Despite the growing body of literature evaluating the use of LLMs in ophthalmology, the performance of a locally deployed domain-specific SLM remains unexplored. Therefore, this study assessed the efficacy of SLM enhanced with RAG technology compared to ChatGPT 4.0 for answering common patient inquiries regarding glaucoma. Glaucoma specialists evaluated the quality of the answers, and the level of readability was assessed using standardized methods.

## Methods

### Study Design

This study was conducted at the Ophthalmology Department of the Bascom Palmer Eye Institute (BPEI) in Miami. Patient information was not included in this study. Between January and February 2024, commonly asked questions related to glaucoma care were queried from reputable online health information outlets, such as the American Glaucoma Society (AGS) and Eye Care Forum, which enables patients to ask questions and receive answers from the American Academy of Ophthalmology (AAO)–affiliated ophthalmologists.

Three fellowship-trained glaucoma specialists refined the first pool of 60 questions extracted from online resources by independently selecting those they considered as frequently asked in a glaucoma outpatient clinic setting. The 35 questions that all specialists considered frequent and common questions from patients with glaucoma were separated for analysis and categorized into 6 domains, such as pathogenesis, risk factors, clinical presentation, diagnosis, treatment and prevention, and prognosis (Multimedia Appendix 1).

### Development of the Ophthalmology-Specific SLM

Our ophthalmology-specific SLM was developed based on the Hugging Face and Haystack algorithms [15,16]. These models serve as a platform for building and deploying NLP models by performing indexing, information retrieval, and question-answering tasks. Specifically, we adopted Mistral 7B, a 7-billion-parameter model, as the SLM [17]. We trained the SLM model using 60 ophthalmology books and 7862 papers from 17 MEDLINE-indexed ophthalmology journals from 2017 to 2023. This process yielded 366,924 snippets, which are succinct excerpts of information extracted from the dataset. These snippets play a crucial role in the operation of RAG, enabling the model to discern the most pertinent information required to address a given question effectively. RAG uses snippets to understand which information is most relevant to answering the specific question asked. These were provided in PDF format to Haystack [16], which processed and split the text into 500-word chunks with 100 words of overlap. These word chunks were converted into model embeddings using the WhereIsAI/UAE-Large-V1 model for training [18] and stored in the Haystack Facebook Artificial Intelligence Similarity Search database. This database is an open-source vector store and search engine that allows for the storage and retrieval of parts of a document relevant to the question being asked. For each question, the 3 most relevant 100-word chunks of text from the reference material were provided alongside the ophthalmology question when prompting the language models. We set the temperature to 0.5, the token limit to 500, and top-p to 1.0. We systematically searched publicly available literature databases, including PubMed and Google Scholar, using the keyword “ophthalmology” to construct the ophthalmology-specific dataset integrated with the RAG system. We prioritized open access documents published in peer-reviewed journals and directly relevant to clinical ophthalmic knowledge.

### Large Language Model

For comparison with LLMs, we used ChatGPT 4.0, developed by OpenAI, a 1.8 trillion-parameter LLM [19]. ChatGPT is a generative artificial intelligence LLM chatbot that interacts with text and engages in human-like interactions [19]. It is built on the GPT architecture and was initially trained on extensive amounts of text from books, papers, and online sources. The model’s training process involves minimizing the difference between the expected and actual words in the dataset, enabling it to produce coherent text based on presented prompts [20,21]. Later versions, such as ChatGPT 4.0, have enhanced their functionalities, with over 1 billion users globally [22]. The performance of the LLM model was assessed using the currently available online version at the time of the study, and only the first response for each question was documented. We used the same inference hyperparameters to ensure comparability with the SLM, with a temperature of 0.5, a token limit of 500, and top-p set to 1.0.

### Prompt Design

Each question was presented to the language models as a standardized prompt, following recent recommendations to maximize the performance of language models [23]. A prompt acts as a clear instruction provided to a language model to generate the desired output, in our case, an answer to a question frequently asked by a patient with glaucoma. The language models were all prompted in a zero-shot fashion, meaning that no examples of questions were provided in the prompt. The prompt was specific and contextual: “Act as a glaucoma specialist during a medical appointment and answer the following question considering it was asked by a patient.” The same prompt was used for the SLM and LLM before each of the 35 selected questions was presented as a stand-alone query. After each query, the conversation was reset to minimize the memory retention bias. All generated responses were formatted as plain text to conceal chatbot-specific features and randomly shuffled before being presented to 3 ophthalmologists for grading of glaucoma.

### Accuracy and Quality Evaluation

Each answer was evaluated by 3 glaucoma specialists (MG, LB, and VVC). The language models’ identities were concealed to prevent bias, and the presentation order was randomized for the graders. Their main task was to individually rate the accuracy of language model responses on a 3-point scale:+1 for responses containing inaccuracies that could significantly mislead patients and potentially cause harm (ie, “poor”);+2 for responses with possible factual errors, but unlikely to mislead or harm patient (“borderline”); and +3 for “good” or error-free responses. Each response’s total quality score was calculated by summing the scores of all 3 graders, with a minimum possible score of 3 and a maximum possible score of 9. In addition, we used a majority consensus approach to obtain an “overall” accuracy rating for each chatbot response, considering the most common rating among the 3 graders. In cases where there was no consensus among graders (ie, each grader provided a different rating), we adopted a stringent approach and assigned the lowest rating. Agreement among graders was evaluated using Fleiss kappa.

### Readability and Quality of Health Information Evaluation

To assess the readability of the chatbot answers, each answer was input into an online readability tool (Readable) [24]. Four readability scales were used, including the Flesch-Kincaid Grade Level, Gunning Fog Index, Coleman-Liau Index, and Simple Measure of Gobbledygook (SMOG) Index. All readability formulas estimate the number of years of education required to fully understand a text. However, each formula uses different equations and variables to calculate it. The Flesch-Kincaid Grade Level focuses on words per sentence and syllables per word. The Gunning Fog Index considers words per sentence and syllables per word. The Coleman-Liau Index measures the average number of letters per 100 words and the average number of sentences per 100 words. The SMOG Index focuses on the number of polysyllabic words in a sample of 30 sentences.

The formula’s output is a number, called the grade level, corresponding to the years of education required to fully understand the text. Content aimed at the public should have a grade level of around 8. Texts above 17 require a graduate-level education for complete comprehension [25].

### Statistical Analysis

Statistical analyses were performed using the Stata Statistical Software Release 18 (StataCorp LLC). The proportions of “Good,” “Borderline,” and “Poor” accuracy ratings were compared between SLM and LLM using a 2-tailed Fisher exact test. The Wilcoxon rank-sum test was used to examine the differences between the 2 language models’ overall answer quality and comprehensiveness scores. Fleiss kappa was calculated to measure interrater agreement. Statistical significance was set at *P*<.05 for all analyses. Post hoc power analysis was performed to assess the observed mean difference in quality scores between the language models. We calculated the standardized effect size based on the observed means and pooled SD and estimated statistical power using a 2-tailed *t* test with an α level of .05.

### Ethical Considerations

In accordance with the Declaration of Helsinki, this study did not involve patients or identifiable private information. Therefore, review and approval by the University of Miami Institutional Review Board were not required.

## Results

A total of 35 frequently asked questions from patients with glaucoma were answered by the LLM and SLM and evaluated by the 3 glaucoma specialists, and a total of 105 gradings were assigned. The interrater agreement, measured by Fleiss κ among graders, was 0.28. The partial agreement rate between graders was 94.3% (99/105). Across the 105 individual accuracy ratings assigned to each model, the LLM had 74% (n=78) of the answers classified as good, 20% (n=21) as borderline, and 6% (n=6) as poor among the graders versus 57% (n=60), 31% (n=33), and 11% (n=12) for the SLM, respectively (*P*=.38). The distribution of quality scores assigned by the graders demonstrated slightly higher central tendency values for the LLM but substantial overlap between models. The median quality score was 8 (IQR 2) for the LLM and 7 (IQR 3) for the SL, indicating greater variability in evaluator scoring. The minimum and maximum observed scores were 5-9 for the LLM and 4–9 for the SLM. No statistically significant difference was observed between the quality scores from SLM (mean 7.4, SD 1.5 points) and LLM (mean 7.9, SD 1.2 points; *P*=.13). Post hoc power analysis indicated that the statistical power to detect this observed difference was 32.9%. Multimedia Appendix 2 details the SLM answers and the references used. Multimedia Appendix 3 shows the answers provided by ChatGPT 4.0.

Table 1 presents an analysis of the consensus-based accuracy ratings overall and across the 6 glaucoma care domains. There was no difference in overall accuracy ratings between the language models (*P*=.38). For each domain, both models performed similarly in all areas. The highest performance by the SLM was in pathogenesis, with 86% (6/7) of the answers graded as “Good,” while the lowest was in treatment and prevention, where 28.5% (2/7) of the answers were graded as “Poor.” Alternatively, LLM’s greatest performing domains were pathogenesis, treatment and prevention, and prognosis. LLM’s worst performance domain was risk factors, where 17% (1/6) of the answers were graded as “Poor.”

**Table 1.** Consensus-based accuracy ratings of natural language models responses across glaucoma care domains.

Domain	Number of questions	Small language model, n (%)			Large language model, n (%)			*P* value
		Poor	Borderline	Good	Poor	Borderline	Good	
Pathogenesis	7	0	1 (14)	6 (86)	1 (14)	0	6 (86)	≥.99
Risk factors	6	1 (17)	2 (33)	3 (50)	1 (17)	1 (17)	4 (66)	≥.99
Clinical presentation	6	1 (17)	1 (17)	4 (66)	0	3 (50)	3 (50)	.54
Diagnosis	2	0	1 (50)	1 (50)	0	1 (50)	1 (50)	≥.99
Treatment and prevention	7	2 (28.5)	3 (44)	2 (28.5)	0	1 (14)	6 (86)	.14
Prognosis	7	0	3 (43)	4 (57)	0	1 (14)	6 (86)	.56
Overall	35	4 (11.55)	11 (31.5)	20 (57)	2 (6)	7 (20)	26 (74)	.38

Table 2 shows the quality scores for each natural language model overall and throughout the 6 glaucoma care domains. The overall quality scores for the SLM and LLM were 258 and 277 (*P*=.13), respectively. The differences in quality scores between all the glaucoma care domains were not statistically significant.

**Table 2.** Consensus-based quality scores of natural language models responses across glaucoma care domains.

Domain	Number of questions	Quality scores		*P* value
		Small language model	Large language model	
Pathogenesis	7	58	56	.62
Risk factors	6	41	46	.40
Clinical presentation	6	46	46	.87
Diagnosis	2	15	14	.68
Treatment and prevention	7	46	58	.09
Prognosis	7	52	57	.45
Overall	35	258	277	.13

Table 3 summarizes the readability scores of the responses for each natural language model. The mean Flesch-Kincaid grade level was 13.2 (SD 3.2) for the SLM and 11.8 (SD 2.2) for the LLM. For the Gunning Fog Index, mean scores were 17.7 (SD 4.3) for the SLM and 14.4 (SD 3.0) for the LLM. The mean results of the Coleman-Liau Index were 14.7 (SD 3.0) for the SLM compared to 12.5 (SD 1.5) for the LLM. The mean scores of the SMOG Index were recorded as 15.98 (SD 2.9) for the SLM and 13.9 (SD 2.1) for the LLM. In all 4 readability classification systems, the SLM had statistically significantly higher scores (*P*<.001).

**Table 3.** Mean readability grade level for small language model and large language model responses^a^.

Readability scores	Flesch-Kincaid grade level, mean (SD)	Gunning fog index, mean (SD)	Coleman-Liau index, mean (SD)	Simple measure of gobbledygook (SMOG) Index, mean (SD)
SLM^b^	13.2 (3.2)	17.7 (4.3)	14.7 (3.0)	15.98 (2.9)
LLM^c^	11.8 (2.2)	14.4 (3.0)	12.2 (1.5)	13.9 (2.1)

a *P*<.001 in all 3 comparisions.

b SLM: small language model.

c LLM: large language model.

## Discussion

### Principal Findings

In this study, we developed and evaluated an SLM trained specifically in ophthalmology to yield clinically relevant information and answer frequently asked questions about glaucoma. The responses provided by our model were as accurate as ChatGPT 4.0, an LLM trained with billions of parameters, as evaluated by glaucoma specialists. To the best of our knowledge, this is the first study to compare the performance of an SLM powered by RAG with ChatGPT 4.0, demonstrating the feasibility of using a local model to answer frequently asked questions about glaucoma and provide references for further reading.

The answers from the SLM developed in this study achieved a mean quality score of 7.4 (SD 1.5) points, which was comparable to the mean quality score of the LLM (7.9, SD 1.2 points out of a total of 9 points; *P*=.13). Moreover, the consensus-based accuracy ratings for the answers of both natural language models were also considered equivalent (*P*=.38). The performance of SLM was also comparable in all 6 glaucoma domains studied, including pathogenesis, risk factors, clinical presentation, diagnosis, treatment and prevention, and prognosis. These results highlight the potential role of SLMs in ophthalmology practice, as they offer a more affordable, adaptable, and straightforward integration into actual ophthalmology clinics. Furthermore, unlike ChatGPT 4.0, which is not open-source and refines its model using user-provided information, SLMs can be trained and operated locally within an institution, significantly reducing the risk of sensitive information leakage, making them a more realistic choice for future integration of natural language models in practical settings [12]. A previous study by Sharir et al [26] estimated the cost of US $80,000 per 1.5 billion parameter model. In this context, training a model such as ChatGPT 4.0 would require US $96,000,000, while an SLM such as the one used in our study would require US $373,000, a more realistic amount for many institutions worldwide [26].

The use of natural language models in artificial intelligence–driven chatbots has increasingly infiltrated daily life [27]. The ability of these models to provide immediate answers across a wide array of inquiries has garnered considerable interest in the health care sector [28-30]. In ophthalmology practice, one of the most relevant applications of natural language models is responding to patient queries commonly encountered in practice [31-33]. Lim et al [32] compared the performance of 3 different LLMs in answering frequent questions about myopia. Using a 3-level grading scale similar to our study (poor, borderline, and good), they reported mean total scores of 8.19 (SD 1.14) for ChatGPT-4.0, 7.35 (SD 1.70) for ChatGPT-3.5, and 7.13 (SD 1.63) for Google Bard. Regarding categorical ratings, 80.6% of ChatGPT-4.0 responses were classified as “good,” compared to 61.3% for ChatGPT-3.5% and 54.8% for Google Bard. Our findings, with mean total scores of 7.9 (SD 1.2) points for the LLM (ChatGPT-4.0) and 7.4 (SD 1.5) points for the ophthalmology-specific SLM, align closely with these previous results. Furthermore, the proportion of responses classified as “good” in our study (78/105, 74% for the LLM and 60/105, 57% for the SLM) is consistent with previously reported results also by Lim et al [32]. While Momenaei et al [33] evaluated ChatGPT 4.0’s ability to address retinal disease queries, responses were considered appropriate in 84.6%, 92%, and 91.7% of the questions concerning retinal detachments, macular holes, and epiretinal membranes, respectively. In both instances, the ChatGPT 4.0 responses were graded by different groups of ophthalmologists as consistently appropriate. Despite these positive results, LLMs, such as ChatGPT, are often expensive, inflexible, and unfeasible to implement in local contexts. Recent advancements in NLP also include multimodal LLMs [34]. For instance, Choi et al [34] successfully used multimodal language models to integrate structured ocular data to calculate safety indicators and predict contraindications in laser vision correction procedures. Their results indicated superior accuracy and flexibility compared to traditional machine learning approaches, underscoring significant clinical potential. Despite these encouraging outcomes, practical challenges remain regarding the broader implementation of such advanced technologies in clinical settings. Specifically, multimodal models often require significant computational resources, entail high costs, and may raise concerns about data security and patient privacy. Thus, while multimodal approaches offer considerable promise, specialized smaller scale models, such as the SLM presented in our study, represent a cheaper and feasible solution for real-world deployment, balancing accuracy, adaptability, cost-efficiency, and local data control.

One major concern of implementing ChatGPT in clinical settings is its lack of ability to provide source citations [35]. Studies have indicated that ChatGPT often provides false references for its generated responses, leading to concerns over response reliability and the risk of inaccuracies [36]. In contrast, the combination of RAG with SLM guarantees the citation of all sources, offering clear evidence for shared information. This ability is a crucial benefit of SLM in clinical contexts, enhancing its utility in delivering reliable, evidence-supported information to patients. Unlike ChatGPT 4.0, which cannot cite references for its responses, SLM equipped with RAG can specify the exact reference and its metadata, including DOI, publication year, and journal name, used to generate a response. The ability to locally deploy domain-specific SLMs with RAG opens several avenues for real-world clinical use. In ophthalmology clinics, SLMs could serve as virtual assistants capable of providing preliminary education to patients, addressing common concerns before or after consultations, and supporting decision-making through curated literature. This could reduce physician workload and improve information retention. These systems could also be embedded in telemedicine platforms or patient portals to enhance access to personalized, trustworthy, and reference-backed content, especially for chronic conditions like glaucoma.

Although our study did not directly compare the models’ responses to responses by human experts, recent evidence suggests that language models may already be approaching human-level performance in natural language generation [37]. A preprint by Jones et al [37] demonstrated that when appropriately prompted to adopt a human persona, state-of-the-art LLMs were judged to be the human more often than real human participants in a controlled 3-party Turing test, effectively passing the original Turing test design. These findings imply that, at least in open-ended conversational tasks, language models may generate responses that are indistinguishable from those of real people. While this supports the plausibility of expert-level performance in patient education tasks, further research is required to compare model-generated content to clinician-authored responses within ophthalmology-specific domains directly.

Previous studies have shown that natural language models often generate grammatically correct responses to common patient inquiries [38]. However, these answers are complex and difficult for the average layperson to understand fully [39]. The American Medical Association recommends that health-related information be communicated at a grade level score of 5-6, which is equivalent to the reading level of fifth- to sixth-graders [40]. Previous research has indicated that information on glaucoma available online is often written at a grade level that is not suitable for health-related information [41-43]. Our analysis revealed that the answers from both LLM and SLM share the same limitation of requiring high-level education to fully understand the answers. In our study, the grade level mean scores, measured by the Flesch-Kincaid Grade Level, the Gunning Fog Index, the Coleman-Liau Index, and the SMOG Index, were 13.2 (SD 3.2), 17.7 (SD 4.3), 14.7 (SD 3.0), and 15.98 (SD 2.9), respectively, for the SLM, and 11.8 (SD 2.2), 14.4 (SD 3.0), 12.5 (SD 1.5), and 13.9 (SD 2.1) for the LLM. The SLM had a statistically significantly higher grade level in all 4 metrics (*P*<.001). This finding is associated with the usage of scientific resources only as the source material for the SLM responses, as this material is written at an academic level.

This study had several limitations. It was conducted with a limited set of questions, focusing solely on a single ophthalmological condition evaluated by a small panel of 3 glaucoma specialists within a single institution. A multicenter evaluation on a larger dataset of questions would offer additional insights into the performance of the SLM powered with RAG versus LLM in answering questions frequently asked by patients with glaucoma. Moreover, this study did not directly assess patient response evaluations. Future studies measuring patients’ opinions on the clarity and quality of the answers could reveal more details regarding using natural language models as a tool for answering glaucoma-related questions. Additionally, the model was not designed exclusively to respond to frequently asked questions about glaucoma but was trained to address ophthalmological inquiries in a broader and more technical context. This approach could have resulted in an underestimation of the SLM’s performance. However, this study stands as proof of concept, and the SLM can be further tailored to specific tasks and other domains in ophthalmology. Furthermore, the post hoc power analysis shows that the sample size of 35 questions provided only 32.9% power to detect the observed difference in quality scores. This indicates a high risk of a type II error, suggesting that the lack of statistical significance may be due to insufficient power rather than equivalence in model performance. Future studies with larger sample sizes are needed to assess potential differences between SLM and LLM performances more robustly. Moreover, the prompt did not contain specific instructions to generate answers to a particular grade level, which could generate more easily understood questions and should be explored by future studies. Finally, this study did not include a direct comparison between the responses generated by the language models and human experts. Future research should evaluate how SLM and LLM outputs compare to clinician-authored answers regarding accuracy, appropriateness, and patient comprehension.

### Conclusion

In conclusion, our study revealed that a specialized SLM may be able to perform similarly to an LLM in answering frequently asked glaucoma questions. However, their answers were unsuitable for health care–related information, as they would be difficult for the average layperson to comprehend. Given their comparable performance to LLMs, high customization potential, ability to provide citations, low cost, and capacity to operate locally without collecting sensitive data, specialized SLMs may present as a realistic option for deploying NLP in real-world ophthalmology clinical settings. Further research is needed to investigate the incorporation of health care–related texts with greater readability into SLMs, as they could be more easily adapted to generate accurate and easy-to-understand answers.

## Supplementary material

10.2196/72101 Multimedia Appendix 1 List of the 35 frequently asked questions from patients with glaucoma used in the study.

10.2196/72101 Multimedia Appendix 2 Small language model answers and the references used.

10.2196/72101 Multimedia Appendix 3 Responses generated by ChatGPT 4.0.

## Abbreviations

AAO: American Academy of Ophthalmology
AGS: American Glaucoma Society
BPEI: Bascom Palmer Eye Institute
LLM: large language model
NLP: natural language processing
RAG: retrieval-augmented generation
SLM: small language model
SMOG: Simple Measure of Gobbledygook
