# Supplementary material for: Performance of a Small Language Model Versus a Large Language Model in Answering Glaucoma Frequently Asked Patient Questions: Development and Usability Study
Source: JMIR AI. 2026 Jan 6;5:e72101. doi: 10.2196/72101 (PMC12772937; doi:10.2196/72101)
Supplement: Multimedia Appendix 1 [file ai-v5-e72101-s001.docx]

**APPENDIX**

List of the 35 frequently asked questions from glaucoma patients used in the study.

1. What is Glaucoma?
2. What parts of the eye are involved in glaucoma?
3. How is intraocular pressure linked to glaucoma?
4. What is intraocular pressure?
5. What is the optic nerve?
6. What is visual field loss?
7. What is included in the ophthalmic examination for glaucoma?
8. What are the primary risk factors for developing glaucoma?
9. Is age a risk factor for glaucoma?
10. What is considered normal eye pressure?
11. Do individuals with elevated eye pressure always develop glaucoma, or can some have elevated eye pressure without glaucoma?
12. Is it possible to prevent glaucoma through diet?
13. Is high myopia a risk factor for glaucoma?
14. What are the typical symptoms of glaucoma?
15. How is glaucoma diagnosed based on symptoms?
16. What are the different types of glaucoma?
17. What is congenital glaucoma?
18. What surgical treatments are available for glaucoma?
19. Is it possible to have glaucoma in your 20s?
20. What diagnostic methods are used to detect glaucoma?
21. How is intraocular pressure measured in a glaucoma diagnosis?
22. What are the available treatment options for glaucoma?
23. Is there a way to prevent glaucoma?
24. How often should I schedule check-ups for my glaucoma?
25. How long does glaucoma treatment typically last?
26. What are the consequences of leaving glaucoma untreated?
27. Is it safe to use glaucoma eye drops when conceiving or during pregnancy?
28. Can using eye drops for glaucoma raise my blood pressure?
29. What is the prognosis for individuals with glaucoma?
30. How can I determine if my glaucoma treatment is effective?
31. What are the potential risks or side effects of glaucoma treatment?
32. Are there additional measures I can take to protect my vision with glaucoma?
33. What can I expect in terms of my vision now and in the future?
34. Is glaucoma hereditary, and what should I tell my family about my condition?
35. If I have glaucoma and need to have cataract surgery, what should my eye pressure be to proceed, and what is the recommended intraocular lens?
